# An Erythrodermic Psoriasis Flare-Up With Staphylococcus Bacteremia Secondary to COVID-19 Infection: A Case Report

**DOI:** 10.7759/cureus.36418

**Published:** 2023-03-20

**Authors:** Qaisar Ali Khan, Tahmina Khan, Michelle R Anthony, Christopher S Farkouh, Parsa Abdi, Faiza Amatul, Hoor ul Ain

**Affiliations:** 1 Internal Medicine, Khyber Teaching Hospital (KTH) Medical Teaching Institution (MTI), Peshawar, PAK; 2 Internal Medicine, Divisional Headquarter Hospital (DHQ) Teaching Hospital Kohat, Kohat, PAK; 3 Pathology, University of Arizona College of Medicine, Tuscon, USA; 4 Dermatology, Rush Medical College, Chicago, USA; 5 Faculty of Medicine, Memorial University of Newfoundland, St. John's, CAN; 6 Dermatology, Mercer University School of Medicine, Macon, USA; 7 Internal Medicine, Jinnah Medical College, Peshawar, PAK

**Keywords:** dermatology case report, psoriasis, staphylococcus bacteremia, covid-19, erythrodermic psoriasis

## Abstract

Erythrodermic psoriasis (EP) is an autoimmune condition commonly manifested as cutaneous lesions, such as well-demarcated, erythematous, scaly plaques, notably on the extensor surfaces but sometimes present on the scalp, palms, and soles. Various triggering events are known to initiate flare-ups of previously well-controlled/dormant EP. In recent literature, the association of acutely exacerbated EP after symptomatic infection with severe acute respiratory syndrome coronavirus 2 (SARS-CoV-2) has been well-described. Here, we present a case of EP with increased flaking and desquamation of the skin of the whole body (most notably on the palms and soles) after three weeks of symptomatic SARS-CoV-2 infection without any other evident trigger. We aim to describe the symptoms as well as the proper management of a patient afflicted with erythrodermic psoriasis in hopes of aiding future clinicians in the prompt diagnosis and treatment of such a patient.

## Introduction

With millions of confirmed cases and a heavy load on healthcare systems, the ongoing coronavirus disease 2019 (COVID-19) pandemic brought on by severe acute respiratory syndrome coronavirus 2 (SARS-CoV-2) has become a significant public health issue globally [1]. Recent studies indicate that COVID-19 may also have dermatological manifestations, such as aggravation of pre-existing skin conditions, in addition to its well-known respiratory manifestations [2]. One such condition that has been found to worsen in some COVID-19 patients is psoriasis, a chronic inflammatory skin disease that affects up to 3% of the population [3]. Erythrodermic psoriasis (EP) is the most aggressive form of psoriasis and has increased morbidity and mortality. Only a few cases of EP flare-ups have been reported in patients who recently had a COVID-19 infection [4]. Dermatologically, diffuse erythema, scaling, and pruritus are the main symptoms of EP. Fever, chills, lethargy, and other systemic symptoms can also be present [5]. Due to the loss of the usual skin barrier function, the skin may also be delicate and vulnerable to blistering and secondary infections leading to substantial protein and fluid loss, electrolyte imbalances, and hypothermia, all of which may necessitate hospitalization and special care management [6,7].

Investigating the prevalence and clinical traits of EP in COVID-19 patients is crucial given the possibility that the condition can worsen or even initiate psoriasis. We aim to enhance the management of this uncommon but severe complication in COVID-19 psoriasis patients as well as identify possible risk factors and therapeutic approaches by presenting a case of EP with increased flaking of the skin of the whole body (most notably on the palms and soles) after three weeks of symptomatic SARS-CoV-2 infection without any other evident trigger.

## Case presentation

**Figure 1:**
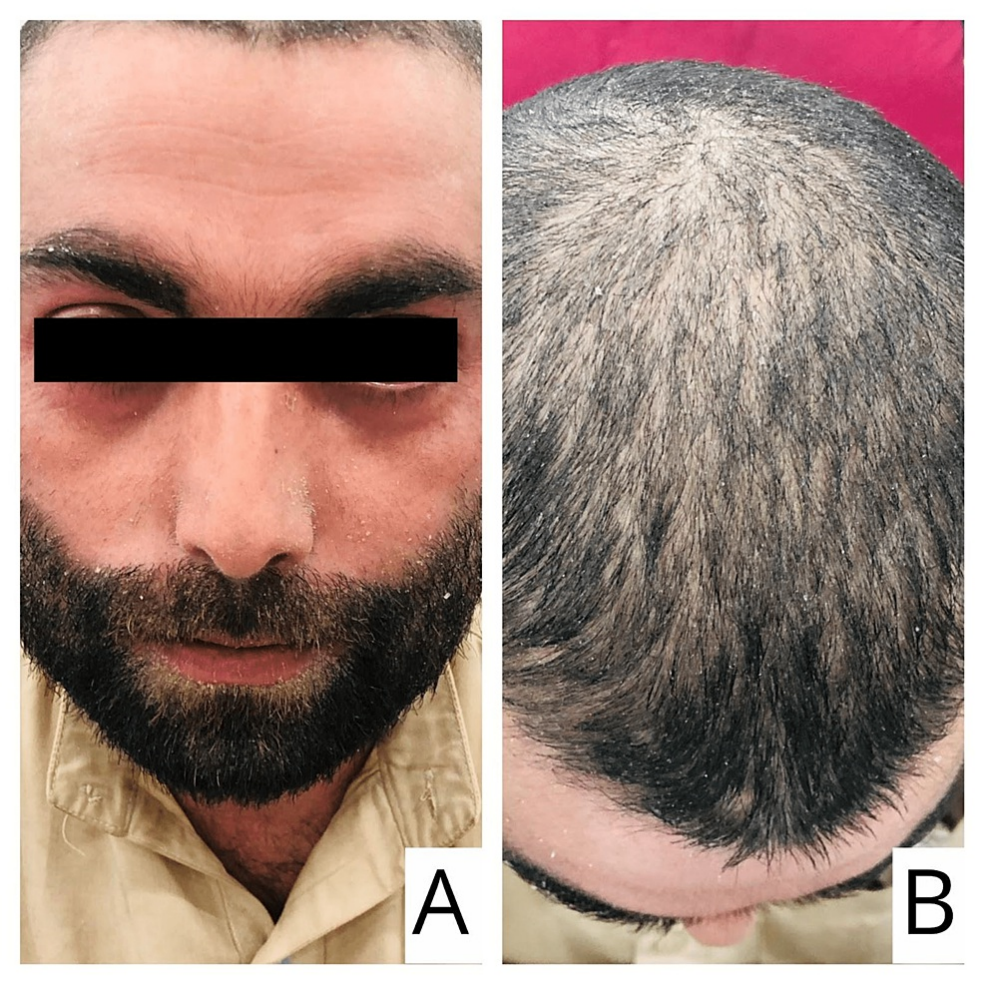
Erythema and skin flaking on the face (A) and scalp (B)

**Figure 2:**
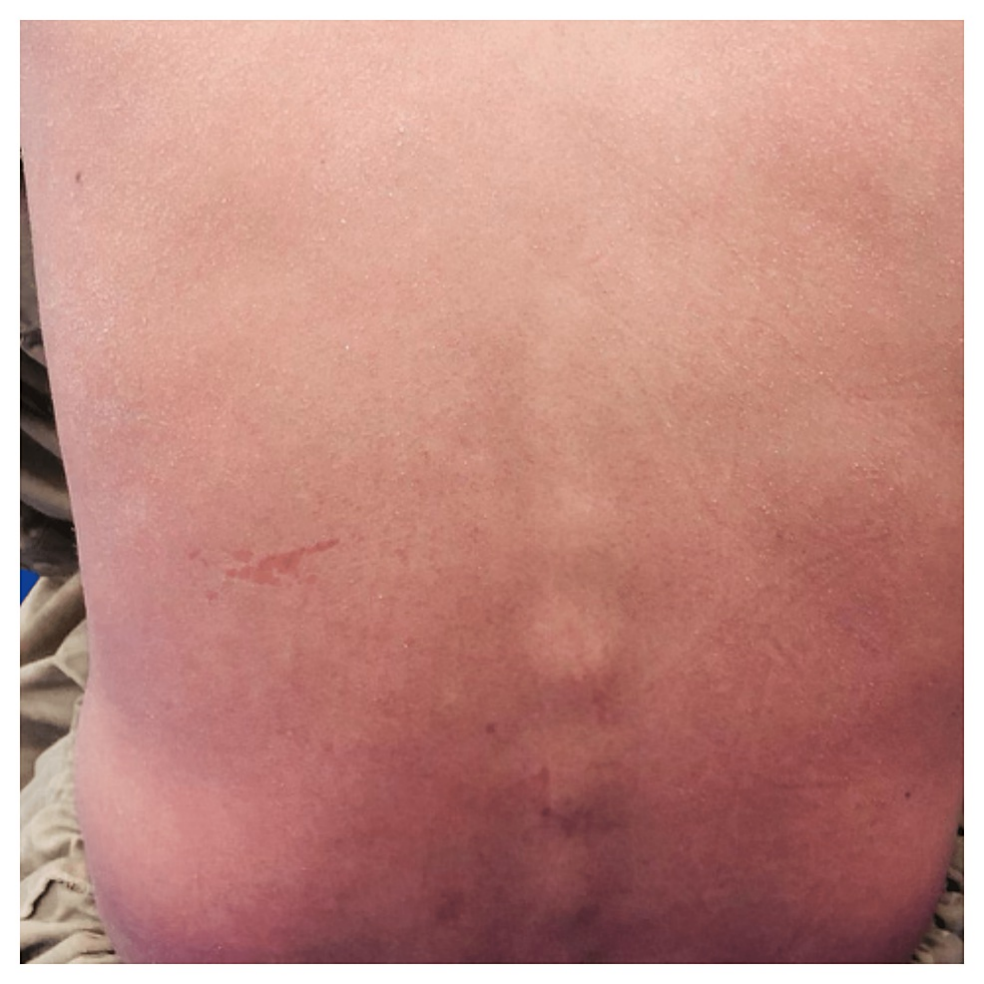
Diffuse erythema and flaking of skin on the lower back of the patient

A 28-year-old male with biopsy-proven plaque psoriasis of 10 years, well-controlled with topical corticosteroid therapy presented to the emergency department with increased erythema, dryness, and desquamation of skin spanning the whole body. The patient did not take systemic therapy for psoriasis in the past. His presentation was associated with low-grade fever and decreased appetite for three days. The symptoms appeared after three weeks of SARS-CoV-2 infection consisting of flu-like symptoms, fever, myalgia, and dry cough, which was diagnosed by COVID-19 polymerase chain reaction (PCR). The patient was quarantined for two weeks and received treatment in the form of vitamin C and paracetamol for SARS-CoV-2 infection. The patient was vitally stable with a blood pressure of 120/80 mmHg, a pulse rate of 88 beats per minute, a respiratory rate of 16 breaths per minute, and a temperature of 100 °F. A dermatological examination revealed diffuse erythema with skin with flaking of the face, scalp, and back (Figure 1 and Figure 2).

**Figure 3:**
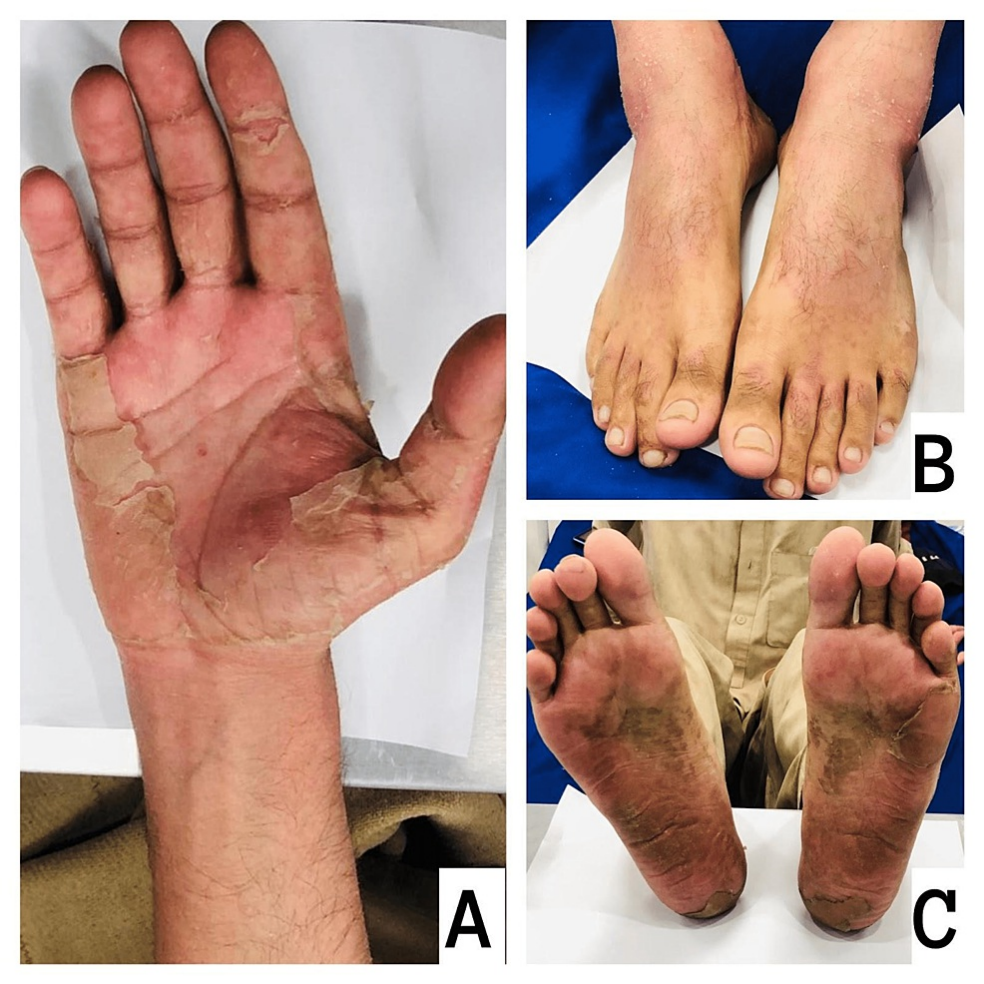
Hyperkeratosis and erythematous skin desquamation with peeling on the palms (A), dorsum of the feet (B), and soles of the feet (C)

Hyperkeratosis and desquamation of the skin were also noted on the palms, soles, and dorsum of the feet (Figure 3). The rest of the bilateral upper and lower limb examination was unremarkable except for the mild erythema. No pitting of the nails was present in both hands and feet.

The other aspects of the systemic examination were unremarkable. Initial laboratory evaluation revealed elevated total leukocyte count with a neutrophilic predominance and a decreased 25-hydroxyvitamin D level as shown in Table 1.

**Table 1:** Patient’s initial laboratory evaluation with normal range references ul: microliter, g/dl: gram per deciliter, Fl: femtoliter, ng/ml: nanogram per milliliter, MCV: mean corpuscular volume

Investigation	Results	Normal range	Unit(s)
Total leukocyte count	14.2	4-11	x10.e 3/μl
Hemoglobin	12.3	12-16	g/dl
MCV	73.4	80-100	fL
Neutrophils	79.4	40-75	%
Lymphocytes	15.2	20-45	%
25 hydroxyvitamin D	20.03	40-100	ng/mL

The patient was admitted to the dermatology unit and a blood culture was sent before the initiation of antibiotic therapy, which revealed Staphylococcus bacteremia. Treatment with IV dexamethasone 8 mg BD, injection ceftriaxone 2 g OD, infusion paracetamol SOS, topical hydrogel, and normal saline soaks was given for one week. The patient improved clinically and was discharged home with oral medication, i.e., tablet Co-amoxiclav 1 gram BD for five days, tablet paracetamol SOS, topical hydrogel, tablet acitretin 25 mg BD, tablet calcium and vitamin D once daily for two months, and tablet methotrexate 2.5 mg every 12 hours in three divided doses. The patient was recommended for weekly follow up and at four weeks, the patient showed improvement in his symptoms, laboratory evaluation remained normal except for decreased vitamin D levels, and the patient was continued with the same treatment.

## Discussion

EP is a severe and rare form of psoriasis vulgaris that affects the entire body and causes systemic inflammation. The skin becomes red, itchy, and uncomfortable, shedding large amounts of scales, and can also cause joint pain, fever, and fatigue. Several factors associated with this disease have been implicated, including interleukin (IL)-4, interleukin-13, and tumor necrosis factor (TNF) alpha [8]. Immunosuppression and immunomodulation treatments have been challenged by the outbreak of COVID-19. In spite of concerns about COVID-19 replication in immunosuppressive drugs, other studies have shown that immunosuppressive drugs are beneficial for preventing drastic immune responses in psoriatic patients infected with the virus [8-10]. The infection of COVID-19 itself provokes immune responses that can progress to cytokine storms, resulting in a state of hyperinflammation in the body that is reminiscent of psoriasis, which also causes hyperinflammation. Other triggering infections for psoriasis include streptococcus, human immune deficiency virus (HIV), Staphylococcus, Helicobacter pylori, Papillomavirus, retroviruses, and some fungal infections such as Mallasezia and candida [11]. COVID-19 infection also provides a nidus for the exacerbation of other immune skin diseases such as pemphigus vulgaris, subacute cutaneous lupus erythematosus, acrodermatitis continua of Hallopeau, systemic sclerosis sine scleroderma, and Sézary syndrome (SS) [12].

The main therapeutic strategy for patients with severe COVID-19 symptoms is to use glucocorticoids; patients with psoriasis, on the other hand, might experience psoriasis flare-ups if they are administered glucocorticoids during COVID-19 infection. Early after steroids are administered, some patients report psoriatic lesions seem to be mitigated, and after the treatment has finished, some patients report experiencing flare-ups of psoriasis. Patients with severe COVID-19 symptoms are usually treated with glucocorticoids as one of their main therapeutic strategies. In psoriatic patients, glucocorticoids administered during COVID-19 infection might affect the patient's psoriasis presentation, as, in some cases, psoriatic lesions appear to be mitigated after the administration of steroids, and some patients describe experiencing flare-ups after completing the course of treatment [13]. The drug hydroxychloroquine (HCQ), used as part of some COVID-19 treatments, has been reported in some cases to worsen psoriasis. It is unclear what causes this effect directly, but some studies attribute it to an elevation of IL-17 in patients taking HCQ [9].

According to literature reviews, COVID-19 does not result in any additional morbidity or mortality in psoriatic patients, and most flare-ups occur in patients who no longer receive biologics and have discontinued taking non-biologic immunosuppressive medications used to treat current EP [14-16]. The current case shows that lack of access to these supplementary medications during COVID-19 causes a flare-up of his long-standing psoriasis.

## Conclusions

COVID-19 infection can provide a nidus for the exacerbation of psoriasis. The severity of the condition depends on the patient's immune response and the treatment received by the patient during the COVID-19 infection. EP is a desquamating condition of the skin that prone the patient to various bacterial infections. Early diagnosis and management are needed for better outcomes.
